# Contrasting patterns of community-weighted mean traits and functional diversity in driving grassland productivity changes under N and P addition

**DOI:** 10.3389/fpls.2023.1145709

**Published:** 2023-08-15

**Authors:** Yuting Yang, Zhifei Chen, Bingcheng Xu, Hossein Ghanizadeh, Wei Li, Chengqin Ding, Ronglei Zhou, Zhongming Wen

**Affiliations:** ^1^ College of Grassland Agriculture, Northwest A&F University, Yangling, Shaanxi, China; ^2^ College of Life Sciences, Guizhou University, Guiyang, Guizhou, China; ^3^ Institute of Soil and Water Conservation, Northwest A&F University, Yangling, Shaanxi, China; ^4^ School of Agriculture and Environment, Massey University, Palmerston North, New Zealand

**Keywords:** leaf traits, root traits, community functional structure, fertilization, semiarid grassland

## Abstract

Fertilization could influence ecosystem structure and functioning through species turnover (ST) and intraspecific trait variation (ITV), especially in nutrient limited ecosystems. To quantify the relative importance of ITV and ST in driving community functional structure and productivity changes under nitrogen (N) and phosphorous (P) addition in semiarid grasslands. In this regard, we conducted a four-year fertilizer addition experiment in a semiarid grassland on the Loess Plateau, China. We examined how fertilization affects species-level leaf and root trait plasticity to evaluate the ability of plants to manifest different levels of traits in response to different N and P addition. Also, we assessed how ITV or ST dominated community-weighted mean (CWM) traits and functional diversity variations and evaluated their effects on grassland productivity. The results showed that the patterns of plasticity varied greatly among different plant species, and leaf and root traits showed coordinated variations following fertilization. Increasing the level of N and P increased CWM_specific leaf area (CWM_SLA), CWM_leaf N concentration (CWM_LN) and CWM_maximum plant height (CWM_H_max_) and ITV predominate these CWM traits variations. As a results, increased CWM_H_max_, CWM_LN and CWM_SLA positively influenced grassland productivity. In contrast, functional divergence decreased with increasing N and P and showed negative relationships with grassland productivity. Our results emphasized that CWM traits and functional diversity contrastingly drive changes in grassland productivity under N and P addition.

## 1 Introduction

In China, the Loess Plateau is a key ecological conservation area, where 42.9% of it accounts for grasslands (Gang et al., 2018; Guo et al., 2022a). Grassland ecosystems play irreplaceable roles in human survival and development due to their ecological (e.g., nutrient cycling, soil and water conservation) and economic (e.g., providing the feed base for grazing livestock) functions (Han et al., 2011; Liu et al., 2021; Guo et al., 2022a). However, massive losses of nitrogen (N) and phosphorus (P) owing to long-term soil erosion have limited the restoration process and ecological functions of grasslands on the Loess Plateau(Liu et al., 2013; Fay et al., 2015; Yang et al., 2022a).

Both N and P, play critical roles in improving community functions and accelerating the grassland restoration process (Rowe et al., 2006; Vitousek et al., 2010; DeSiervo et al., 2023). In the past decades, some studies have looked into how fertilization affects species interaction and biodiversity to understand the ecological functions of grasslands under various nutrient levels in the Loess Plateau and to develop management practices to improve the restoration process (Wang et al., 2014; Chen et al., 2021; Yang et al., 2022b). However, most researchers primarily assessed the impact of various fertilization levels on grassland ecosystem functioning at the community level, while they did not consider their relationship with the species composition changes and the ecophysiological responses at the individual species level characterized by functional traits (Stevens, 2016; Zhang et al., 2018). Investigating the impacts exerted by plant species can further our understanding of the response of grassland ecosystem functions under nutrient addition. Because the response of ecosystems to anthropogenic disturbances and environmental changes starts with the different responses of individual plant species, and subsequently, it scales up to the community level(Lavorel and Garnier, 2002).

At species level, plants can improve their adaptability to environmental changes through trait plasticity (Turcotte and Levine, 2016). Trait plasticity is the ability to manifest different levels of specific traits, enabling plants to cope with various environmental constraints (Funk et al., 2017). Trait plasticity also determines species-specific responses to nutrient addition (Kichenin et al., 2013). However, the direction and magnitude of plasticity differ among species (Lin et al., 2020). Generally, aboveground (i.e. shoot) and belowground (i.e. root system) traits respond differently to environmental changes (Weemstra et al., 2016). While leaf traits are tightly related to photosynthetic function and plant production, root traits determine the nutrient acquisition strategies of plants (Funk et al., 2017; Caplan et al., 2019). Nevertheless, coordination between leaf and root traits plays pivotal roles in forming adaptation strategies to environmental changes (Faucon et al., 2017). Yet, few studies explored the coordination between leaf and root traits in response to nutrient addition in grassland ecosystems on the Loess Plateau.

At community level, environmental changes such as nutrient addition can influence community functional structure through intraspecific trait variation (ITV), species turnover (ST), or both depending on the intensity of changes. ITV represents contribution to the overall functional trait response to environmental changes. ST represents the change of species composition (Albert et al., 2010). Under low-intensity environmental changes, community-level traits may respond through ITV, with no noticeable changes in species compositions (Kichenin et al., 2013). While under high-intensity environmental changes, variation in community-level traits is attributed to ST (Kichenin et al., 2013). ITV and ST play essential roles in grassland ecosystems in shaping the community functional structure under nutrient addition (Read et al., 2017). However, their effects on the community functional structure vary with the studied traits and experimental duration (Zhou et al., 2018). Alterations in the community functional structure are usually determined by community-weighted mean traits (CWM, mass-ratio hypothesis) (Grime, 1998) and functional diversity (FD, diversity hypothesis) (Diaz et al., 2007; Mao et al., 2017). CWM traits focus on dominant species that predominantly determine the responses of community functional structure to nutrient addition (Grime, 1998). While FD describes the functional trait diversity within a species community, and often expresses by functional evenness (FEve), functional dispersion (FDis), functional divergence (FDiv), and Rao’s quadratic entropy (RaoQ) (**Table S1**) (Petchey and Gaston, 2006). Given the role of ITV and ST in ecosystem function, it is crucial to understand the relative contribution of both factors in grassland community functional structure under nutrient addition. Yet, few researchers have assessed if alterations in the community functional structure following nutrient addition are governed by ITV or ST in semiarid grasslands.

Several studies have shown that species functional traits predominantly regulate ecosystem functioning (Wellstein et al., 2011; Chen et al., 2023). Community-level functional traits better explain variations in ecosystem functioning than species based matrices (Roscher et al., 2012). When community functional structure is dominated by ST, the composition of community will shift towards species with dominant functional traits (Smith et al., 2009), leading to alterations in grassland productivity (Isbell et al., 2013). Given that, grassland productivity is a key indicator of grassland ecosystem functioning (Schaub et al., 2020).

Nutrient addition caused species loss and trait divergence, resulting in variation in community functional structure, further affecting grassland productivity (Niu et al., 2014). Hence, a critical factor in developing theoretical support for grassland management is to determine components of community functional structures that affect the variations in ecosystem functioning following nutrient addition. In view of this, a four-year fertilizer (i.e., N and P) addition experiment was conducted on a semiarid grassland to (1) quantify the responses of leaf and root trait plasticity (i.e., species-level) and community functional structures (i.e., community-level) to N and P addition (2) quantify the contribution of ST and ITV in driving the community functional structure in response to N and P addition, and (3) assess the effects of variations in community functional structure on grassland productivity under N and P addition.

## 2 Materials and methods

### 2.1 Study area

**Figure 1:**
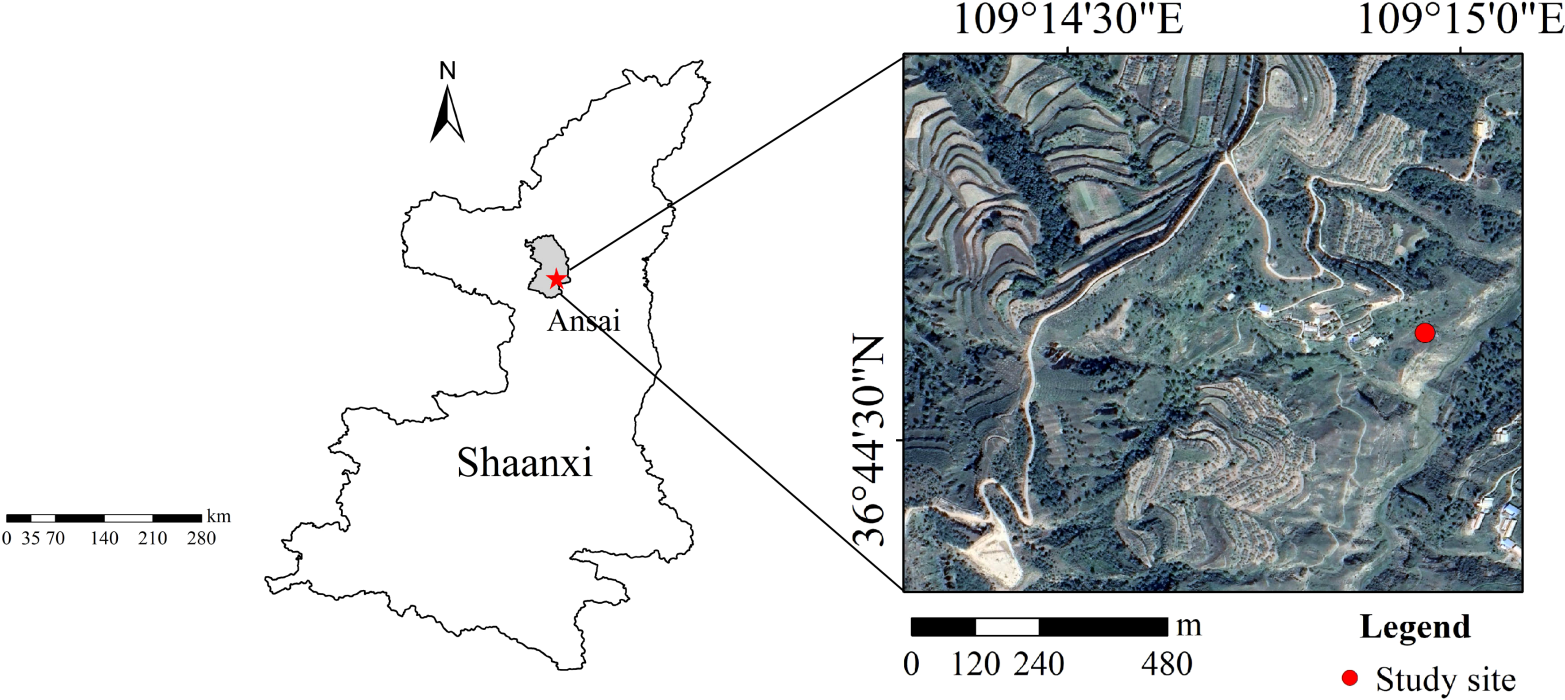
Geographical location of the study site.

The study was conducted in a semiarid grassland in Shaanxi Province, China, with an altitude of 1010–1431 m, an average temperature of 8.8°C and average annual rainfall of 528.8 mm (**Figure 1**). The soil type is loamy loess soil, and the landform is a typical hilly-gullied landscape (Guo et al., 2022b). The soil total N and P of the study area are 0.60 and 0.58 g kg^-1^, respectively. For this research, a 600 m^2^ area of the grassland was fenced to avoid grazing disturbance (**Table S2**). There was a total of 13 species in the grassland community before N and P additions in 2017. The relative abundance of common species were *Bothriochloa ischaemum* (L.) Keng (27.30%), *Artemisia sacrorum* Ledeb. (11.70%), *Lespedeza davurica* (Laxmann) Schindler (11.70%), *Stipa bungeana* Trin. (12.67%), *Potentilla tanacetifolia* Willd. ex D.F.K.Schltdl. (16.36%), and *Artemisia scoparia* (Waldst. & Kit.) Pamp (5.39%). The coverage and aboveground biomass of the grassland community were 41% and 118.7 g m^-2^ before N and P additions in 2017 (**Table S3**).

### 2.2 Experimental design

The research was initiated in late August 2017. The experimental design was split-plot with twelve main plots (4 × 4 m) and four subplots (2 × 2 m). Each main plot was laid out in a randomized block design with three replicates. A 1.5-m buffer zone was allocated between adjacent blocks. Four main plots were randomly assigned to four levels of N, namely 0, 25, 50, and 100 kg N ha^‐1^ yr^‐1^(hereinafter referred to as N0, N25, N50, and N100, respectively). Moreover, four subplots per main plot were randomly assigned to four levels of P, namely 0, 20, 40, and 80 kg P_2_O_5_ ha^‐1^ yr^-1^ (hereinafter referred to as P0, P20, P40, and P80, respectively) (**Figure S1**). The lowest N addition level (N25) was applied according to the atmospheric N deposition level (about 21.76 kg N ha^-1^ yr^-1^) on the Loess Plateau (Liang et al., 2016). N50 and N100 treatments were about twice and four times of atmospheric N deposition level to estimate further consequences of N deposition. The P addition levels were applied according to the proper P addition treatments in the experiment of promoting grassland restoration based on our previous study. N and P fertilizers were applied in calcium ammonium nitrate and triple superphosphate, respectively. Fertilization was applied manually to each subplot every year at the beginning of the growing season before rainfall on June 4, 2017, May 21, 2018, June 13, 2019 and June 11, 2020, respectively. Two separate 1 × 1 m fixed quadrats were established randomly in each subplot for community survey (i.e., structure and function) and measuring functional traits.

### 2.3 Community survey and trait measurements

Plant maximum plant height (H_max_), abundance, and coverage were measured in August 21, 2020 in each 1×1 m fixed quadrat. The aboveground material of all plant species within each quadrat was cut, oven-dried at 80°C, and weighed. The community productivity was estimated as the sum of the harvested aboveground biomass. Relative biomass of each species was the aboveground biomass of each species/community aboveground biomass.

Plant functional traits were evaluated using 2–3 fully mature individuals for each dominant species (i.e., those species account for at least 80% of community biomass) in each fixed quadrat. 10-15 mature and fully expanded sun-exposed leaves per individual were chosen for measuring leaf traits, and 10-15 fine roots (diameter< 2 mm) of the same individuals from which we collected leaves were chosen for measuring fine root traits. Functional traits of leaves and roots were measured according to standard protocols (Pérez-Harguindeguy et al., 2013). The leaf and root samples were placed in sealed plastic bags and stored in a chiller until they were weighed and scanned. We used a digital caliper to measure leaf thickness at the midpoint of leaves while avoiding the major veins. We then scanned the leaf and root samples using a portable scanner (CanoScan LiDE 120) and measured the leaf area using the ImageJ software (http://imagej.nih.gov/ij/). Each fine root image was processed using the WINRHIZO software (Regent Instruments Inc., Quebec City, Canada) to determine the root length and root surface area. All samples were oven-dried for 48 h (65°C) to determine the leaf and root dry mass. We then calculated the specific leaf area (SLA; cm^2^ g^-1^) as leaf area/leaf dry mass and the specific root length (SRL; cm g^-1^) as root length/root dry mass. Leaf tissue density (LTD; g cm^-3^) was calculated as leaf dry mass/leaf volume, and leaf volume was calculated as leaf area × leaf thickness. The specific root surface area (SRA; cm^2^ g^-1^) was calculated as root surface area/root dry mass. Finally, the leaves and fine roots of each species were ground and passed through a 0.25-mm sieve. The sieved samples were used to measure the leaf and root N and P concentrations (LN, LP, RN, and RP, respectively; g kg^-1^). LN and RN were measured with an auto-Kjeldahl instrument (Kjektec System 2300 Distilling Unit, Foss, Sweden). LP and RP were determined using the molybdenum–antimony colorimetric method.

### 2.4 Statistical analysis

Plant trait plasticity was evaluated using the plasticity index (PI). Under fertilization addition, a positive PI indicates that trait values are higher than that of the control treatment (Lin et al., 2020). The PI was calculated following Godoy et al. (2011):


(1) $$\begin{array}{c} \text{PI =}\frac{\text{Mean (fertilization) - Mean (N0P0)}}{\text{Max (Mean (fertilization), Mean (N0P0))}}\; \end{array}$$

where ‘Mean (fertilization)’ and ‘Mean (N0P0)’ denote the mean functional traits of species under nutrient addition and N0P0 treatments, respectively, ‘Max [Mean (fertilization), Mean (N0P0)]’ represents the maximum mean values of the assessed traits in each treatment.

To determine the community functional structure, The ‘dbFD’ function in the *FD* package was used to calculate the CWM traits and FD in R 4.1.3(R Development Core Team). The contribution of ST and ITV in explaining variations in functional diversity and CWM traits was determined based on the method proposed by Lepš et al. (2011), which is based on the sum of squares decomposition method. The total Sum of Squares (SS) was the total variation of ITV, ST and covariation. Detailed information on this method is provided in the supplementary file.

The effects of N and P addition on CWM traits and FD were analyzed using an analysis of variance (ANOVA). The least significant difference (LSD) criterion was used for *post hoc* multiple comparisons of significant differences between different N and P treatments. The ANOVA was performed using GenStat version 18.0 (VSN International Ltd., Rothamsted, UK).

A principal component analysis (PCA) was conducted to correlate functional traits and relative biomass using CANOCO 5.0 (ter Braak and Smilauer, 2012). In order to evaluate the effects of the changes of different CWM traits and different FD caused by different N and P addition on community productivity, redundancy analysis (RDA) analysis was conducted to evaluate the effect of the changes of different CWM traits and different FD on community productivity via the “vegan” package in R 4.1.3. The relative contribution of CWM traits and different FD to community productivity were estimated on the basis of hierarchical partitioning via the “rdacca.hp” package in R 4.1.3 (Lai et al., 2022). Eventually, SEM (structural equation model) was constructed using Amos 24.0 (Amos Development Co., Greene, Maine, USA) to evaluate the relative importance and hypothetical paths of the community functional structure exhibiting significant independent effects and regulating community productivity. Root mean square error of approximation (RMSEA) and a chi-square (χ^2^) test with the associated probability were adopted to evaluate model fitness. The model fit was deemed acceptable when 0 ≤ χ^2^/df ≤ 2, 0 ≤ RMSEA ≤ 0.1 and χ^2^ and RMSEA values were nonsignificant (*p*>0.05).

## 3 Results

### 3.1 Trait plasticity

It was noted that N addition resulted in significant impacts on all functional traits except for SLA, SRA, RN and RP (*p*< 0.05; **Table S4**). LP, LN: LP, LTD, RP and RN: RP were the only functional traits significantly impacted following P addition (*p*<0.05; **Table S4**).

**Figure 2:**
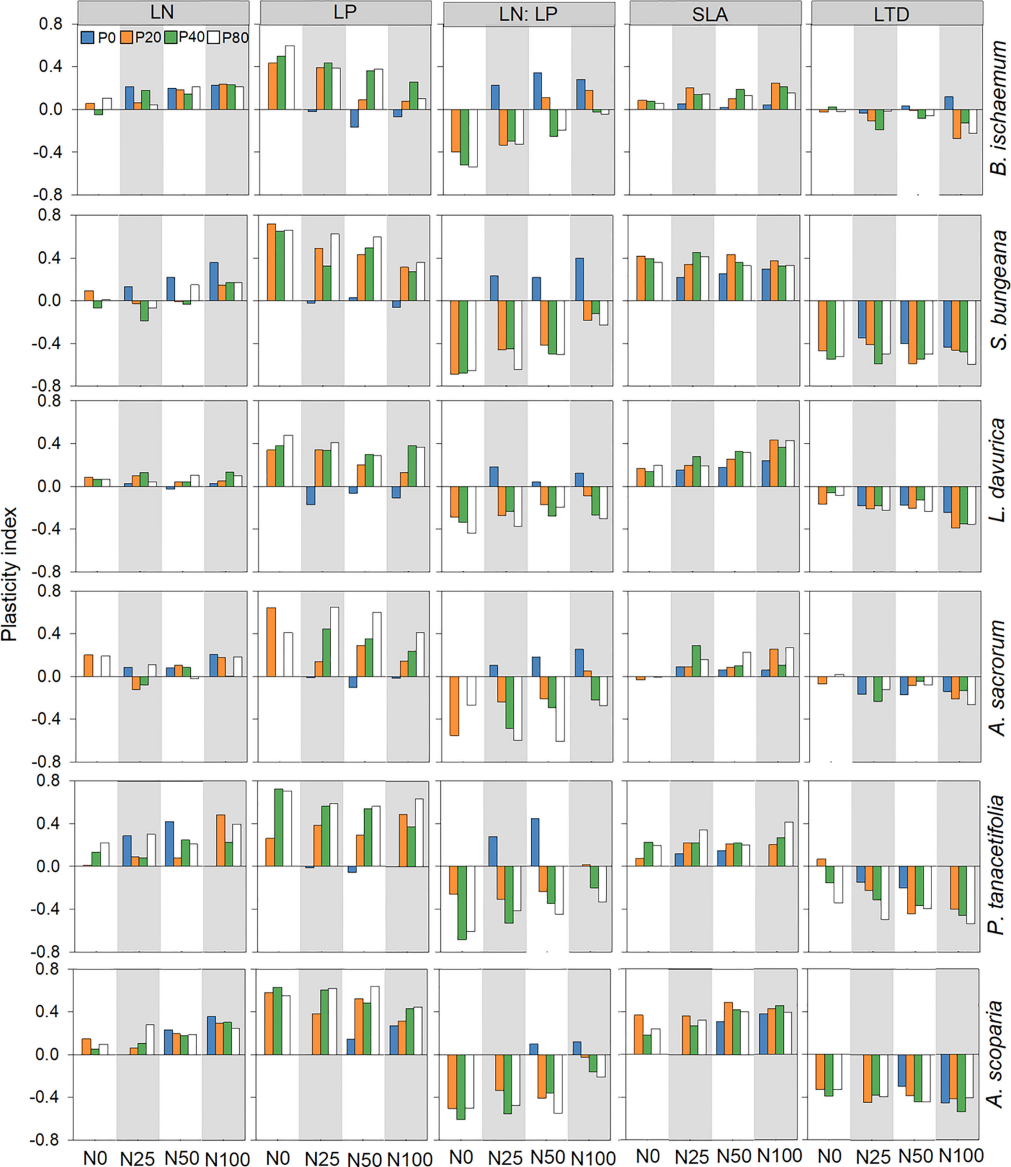
Leaf trait plasticity index of plant species following different levels of N and P addition. leaf P concentration (LP) Leaf N concentration (LN); leaf N:P ratio (LN: LP); specific leaf area (SLA); leaf tissue density (LTD).

The PI of LN was greater than zero in all treatments for *B. ischaemum* (except for N0P40), *P. tanacetifolia*, and *A. scoparia*. The PI of LP for *A. scoparia* was greater than zero in all treatments, while the PI of LP was negative for the other five species when they were treated with N addition only. The PI of LN: LP was only higher than zero for all species treated only with N. The PI of SLA was greater than zero for all six species in all treatments, except for *A. sacrorum* treated with P-only (**Figure 2**).

**Figure 3:**
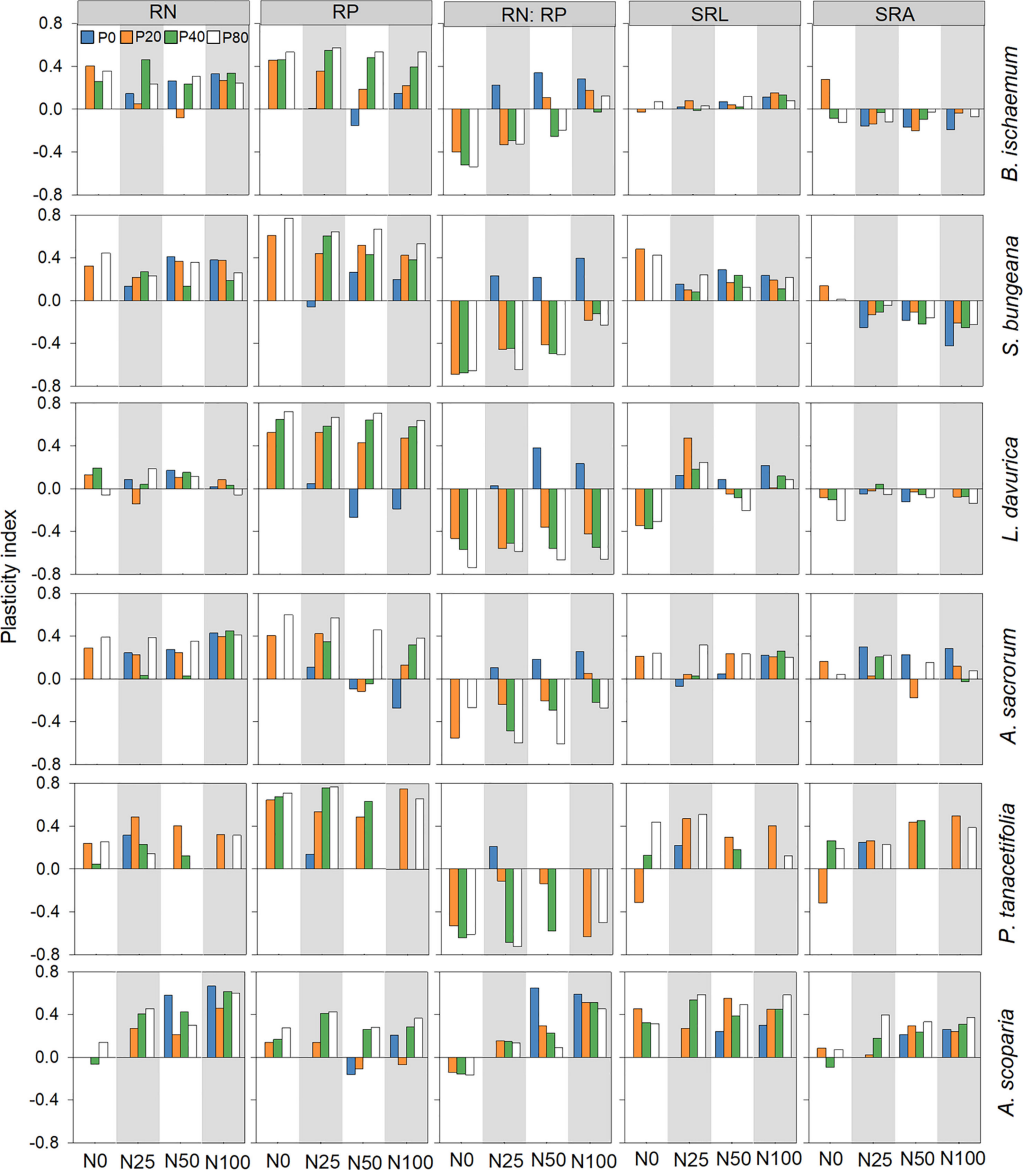
Root trait plasticity index of plant species following different levels of N and P addition. Root N concentration (RN); root P concentration (RP); root N:P ratio (RN: RP); specific root length (SRL); specific root surface area (SRA).

The PI of RN was greater than zero for all species and treatment combinations except for *B. ischaemum* treated with N50P20, *L. davurica* treated with N0P80, N25P20, and N100P80, and *A. scoparia* treated with N0P40. Similarly, the PI of RP was greater than zero for most of the species and treatment combinations. However, the PI of RN: RP was generally higher than zero for all species treated only with N. The PI of SRL was greater than zero for all species and treatment combinations except for *L. davurica* treated with P0 and various levels of P in combination with N50 and *P. tanacetifolia* treated with N0P20 (**Figure 3**).

All six species showed substantial variations in PI. For instance, the PI of SLA of *B. ischaemum* was significantly lower than *S. bungeana* and *A. scoparia* (*p*<0.05; **Figure 2**; **Table S5**). It was also noted that the plasticity in LTD of *S. bungeana* exhibited significantly lower plasticity than other species for (*p*<0.05; **Figure 2**; **Table S5**), and the PI of SRL was significantly greater for *S. bungeana* and *A. scoparia* than *B. ischaemum* (*p*<0.05; **Figure 3**; **Table S5**).

### 3.2 Relative biomass and functional traits

**Figure 4:**
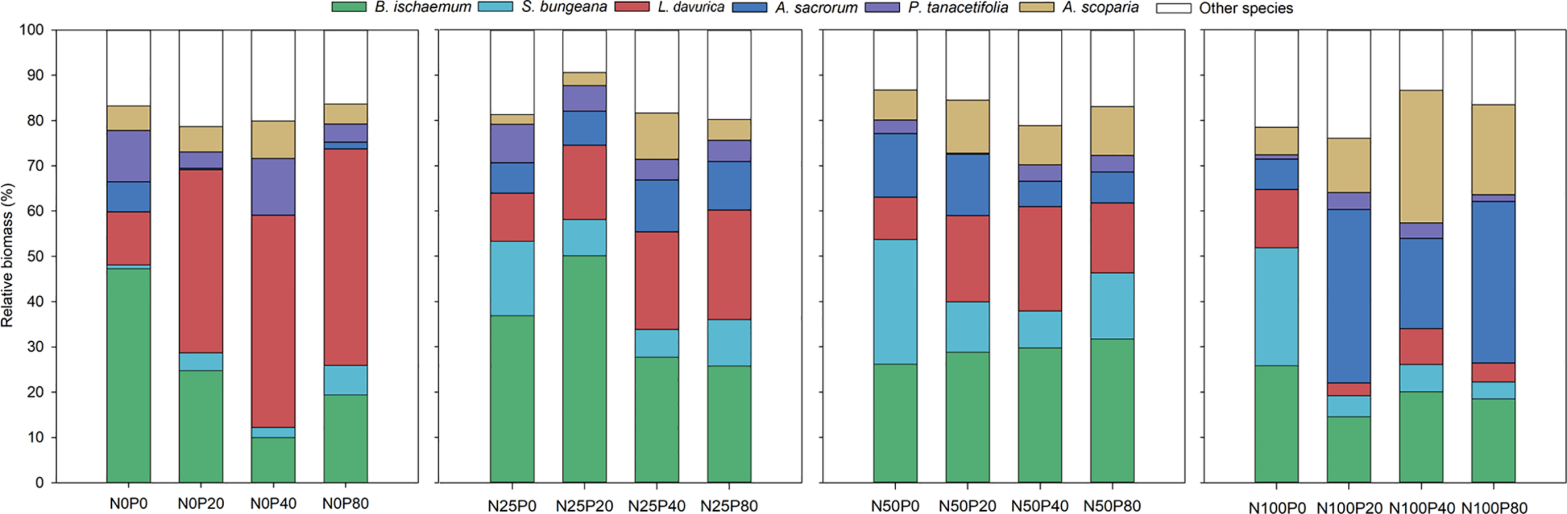
Relative biomass of plant species following different levels of N and P addition.

The relative biomass of *B. ischaemum* was significantly higher in N25 and N50 treatments than that recorded for the N100 treatment, regardless of the level of P (*p*<0.05; **Figure 4**; **Table S6**). N and P interaction showed significant effects on the relative biomass of *L. davurica*, it was significantly lower in treatments involving N in combination with various P levels than in P-only treatments (*p*<0.05). In contrast, a combination of N and P resulted in significantly greater relative biomass in *A. sacrorum* compared to those treatments involving only one of these nutrient elements (*p*<0.05). The relative biomass of *A. scoparia* was significantly higher in treatments involving N100 with various P levels than in the P-only treatment (*p*<0.05; **Figure 4**; **Table S6**).

**Figure 5:**
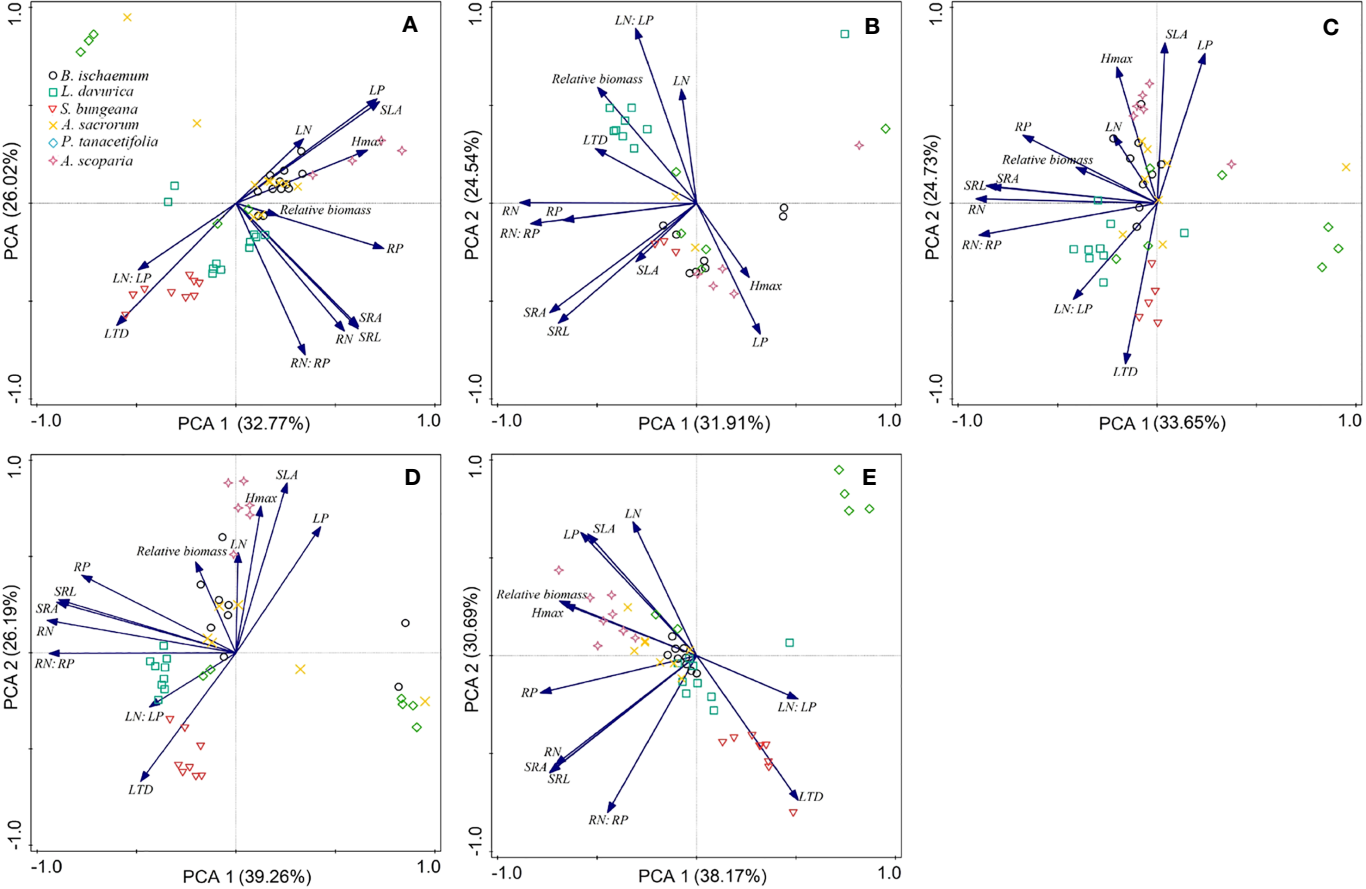
Principal components analysis (PCA) of functional traits and relative biomass of the six species after N and P addition. **(A)**: only N; **(B)**: only P; **(C)**: N25 in combination with all levels of P; **(D)**: N50 in combination with all levels of P; **(E)**: N100 in combination with all levels of P.

Different relationships between relative biomass and functional traits were recorded following different levels of N and P addition (**Figure 5**). The increase in the relative biomass was positively related to RP, H_max_ and SLA in the N-only treatment (**Figure 5A**). The higher relative biomass was primarily due to an increase in LN: LP in the P-only treatment (**Figure 5B**). The higher relative biomass was correlated with high RP, SRL, and SRA in treatments involving N25 combined with P (**Figure 5E**). The increase in relative biomass was positively associated with RP, H_max_ and SLA in N50 combined with P (**Figure 5D**). The higher relative biomass was mainly due to an increase in H_max_ treated with various P levels in combination with N100 (**Figure 5E**).

### 3.3 Community functional structure

**Figure 6:**
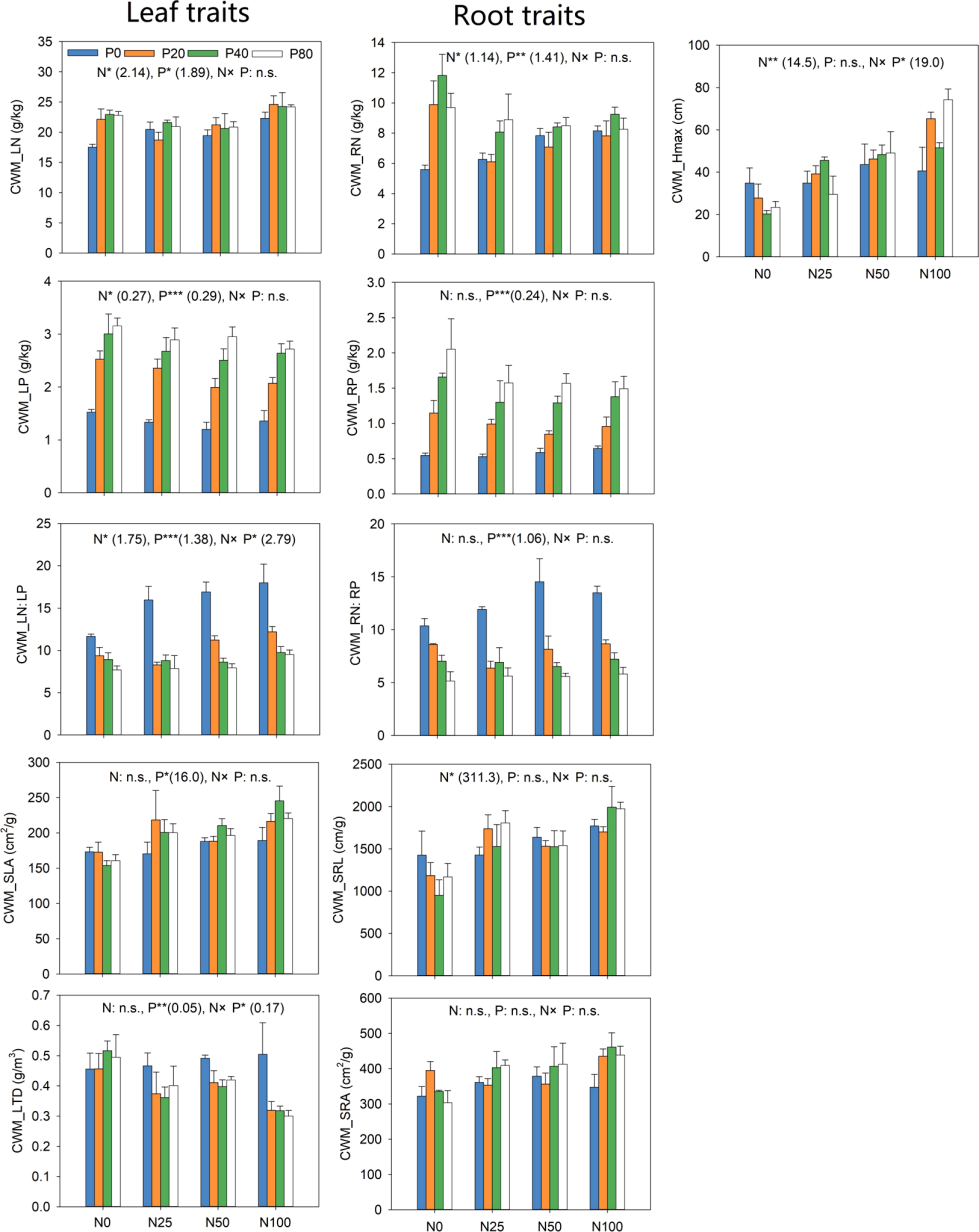
Response of community-weighted mean (CWM) traits to N and P addition. The numbers in brackets are the values of Least significant difference (LSD). *p<0.05; **p<0.01; ***p<0.001; n.s. not significant.

Adding N and P led to divergent CWM values for leaf traits and H_max_ (**Figure 6**). The results revealed that the effects of N addition were significant for CWM_LN, CWM_LP, CWM_LN: LP, and CWM_H_max_ (*p*<0.05), while P addition significantly impacted all leaf traits (*p*<0.05). The interaction between N and P addition significantly impacted CWM_LN: LP, CWM_LTD and CWM_H_max_ (*p*< 0.05). CWM LN increased significantly after treatment with N or P alone than N0P0. Increasing level of P addition significantly promoted CWM_LP. CWM_SLA promoted significantly under all P levels as compared with P0. However, the impact of P addition on CWM_SLA was more pronounced at the highest level of N. CWM_H_max_ increased significantly with increasing the level of N, with P addition synergizing the positive impact of N (**Figure 6**).

N addition only had significant effects on CWM_RN and CWM_SRL (*p*< 0.05). P addition significantly impacted CWM_RN, CWM_RP and CWM_RN: RP (*p*< 0.05). However, N addition only significantly decreased CWM_RN (**Figure 6**). All N levels promoted CWM_SRL significantly compared with N0. All P levels significantly enhanced CWM_RP. In contrast, increasing the level of P significantly reduced CWM_RN: RP (**Figure 6**).

**Figure 7:**
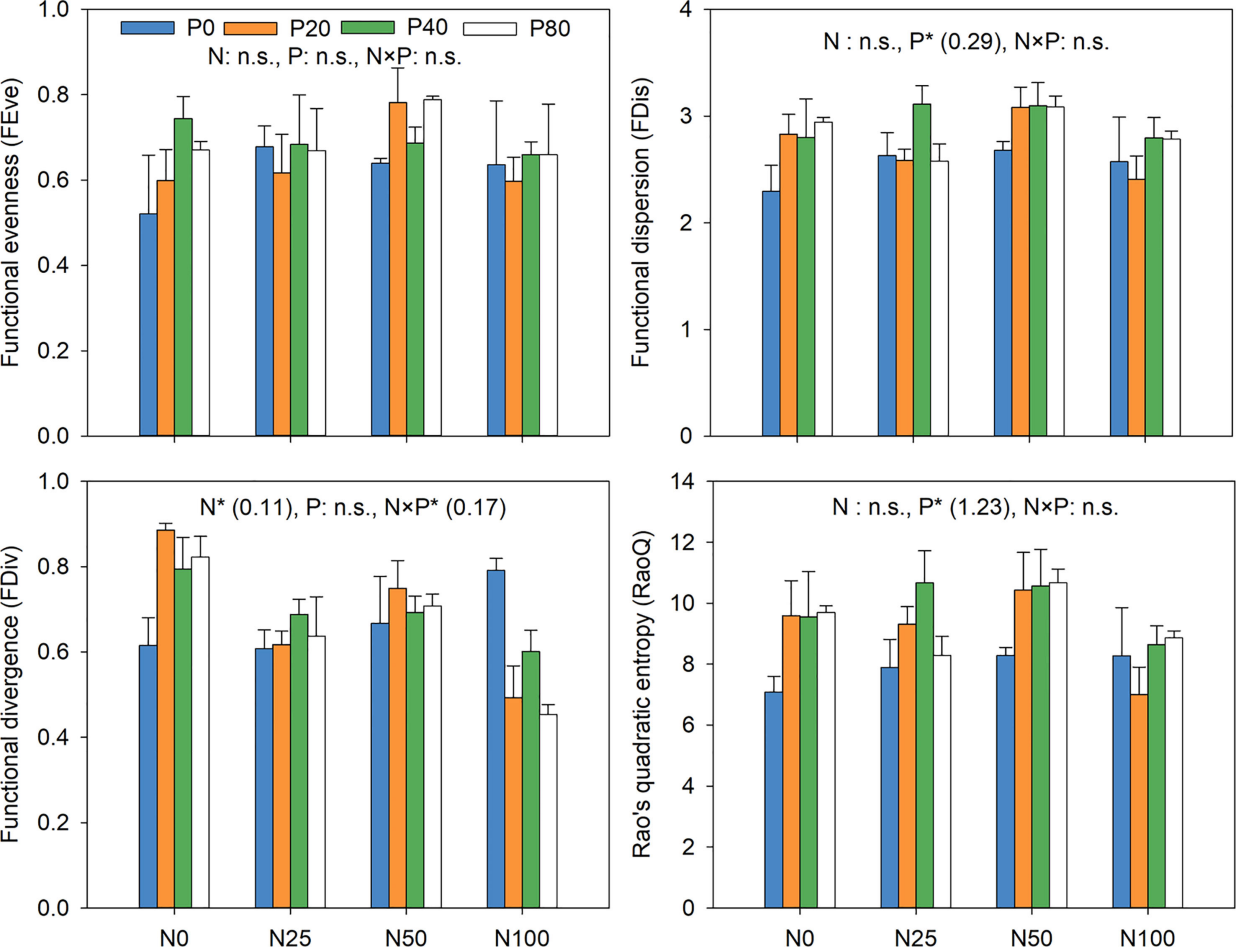
Response of functional diversity to N and P addition. Inset numbers in brackets show the values of Least significant difference (LSD). *p<0.05. n.s. not significant.

N addition had a significant effect on FDiv (*p*<0.05; **Figure 7**), while the addition of P significantly impacted RaoQ and FDis (*p*<0.05). N and P interactions greatly affected FDiv (*p*<0.05). The greatest and lowest values for RaoQ and FDis at all P levels were recorded at N50 and N100, respectively. FDiv was generally lower in all N levels compared to the N0P0, with P addition exacerbating the reduction, especially at N100 (**Figure 7**).

### 3.4 Relative contribution of variability explained by ST versus ITV

**Figure 8:**
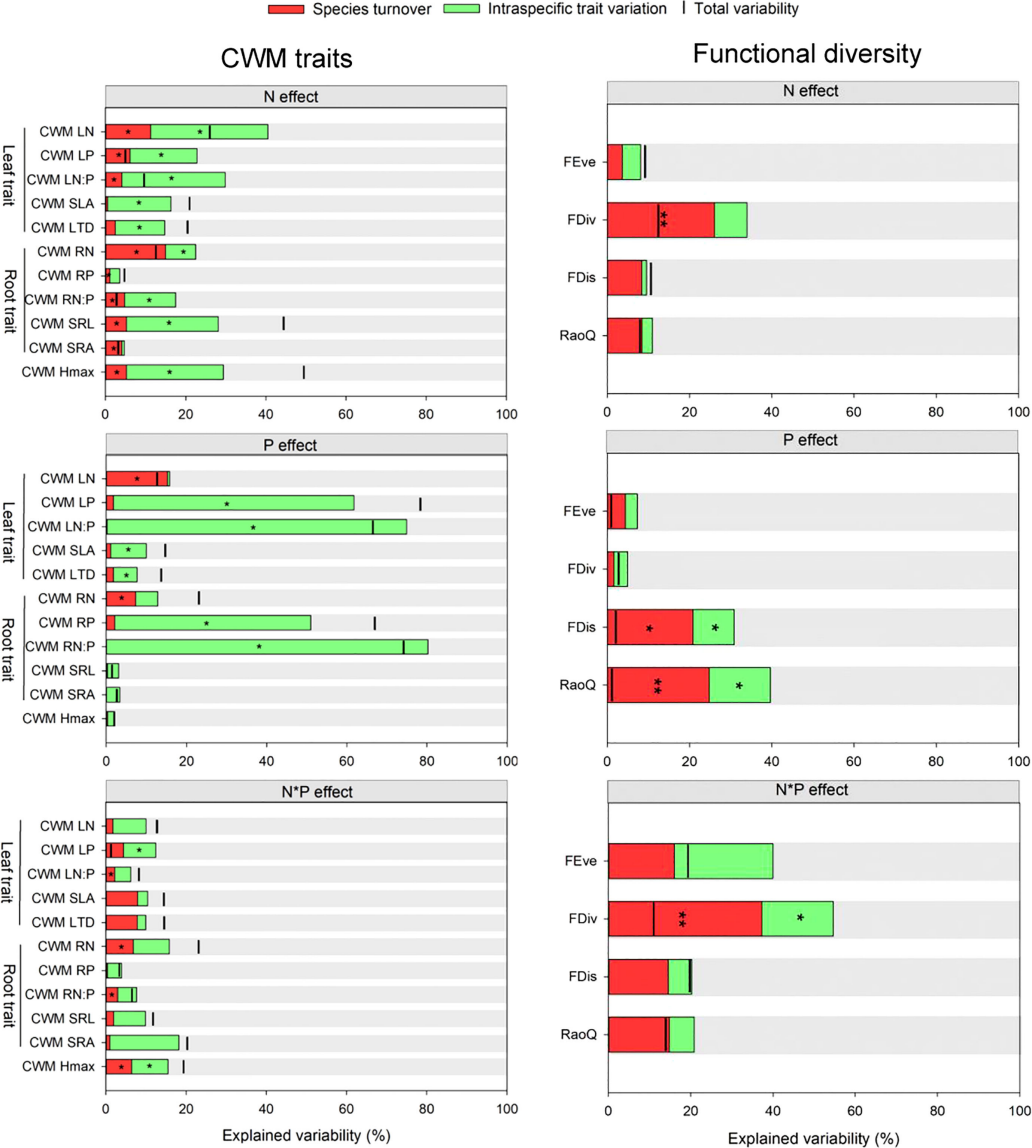
Variability in community-weighted mean (CWM) trait values based on intraspecific trait variation and species turnover. The distance between the bar and the top of the column represents covariation effects. Significant effect is indicated using * and ** at 5% and 1% level of probability.

Under N addition, ITV significantly contributed to the variation in CWM_SLA (16%) and CWM_LTD (12%). Both ST and ITV significantly contributed to variations of CWM_LN, CWM_LP, CWM_LN: LP, and CWM_H_max_ under N addition, though these variations were primarily driven by ITV (16-29%). positive covariance effects between ST and ITV were found for CWM_SLA, CWM_LTD and CWM_H_max_. Under P addition, ST explained more variations in CWM_LN (15%), while the contribution of ITV to the variations in CWM_LP (60%), CWM_LN: LP (75%), CWM_SLA (9%) and CWM_LTD (6%) was more significant. Covariance effects between ITV and ST were only negative for CWM_LN and CWM_LN: LP under P addition. Under N and P interactive effects. Covariance effects between ST and ITV were positive for CWM_Hmax and all CWM leaf trait values (except for CWM_LP) under the interactive effects of N and P addition (**Figure 8**).

ITV and ST had significant impacts on all CWM values for root traits except for CWM_RP and CWM_SRA under N addition. Under P addition, ITV contributed significantly to changes in CWM_RP (49%) and CWM_RN: RP (80%). Positive covariance effects between ITV and ST were only found for CWM_RP (**Figure 8**). ST had significant impacts on FDiv under N addition. ITV and ST significantly contributed to FDis and RaoQ under P addition, and ST primarily drove them. Under N and P addition interactive effects, ST significantly explained variability in FDiv (**Figure 8**).

### 3.5 Linkages between grassland productivity and community functional structure

**Figure 9:**
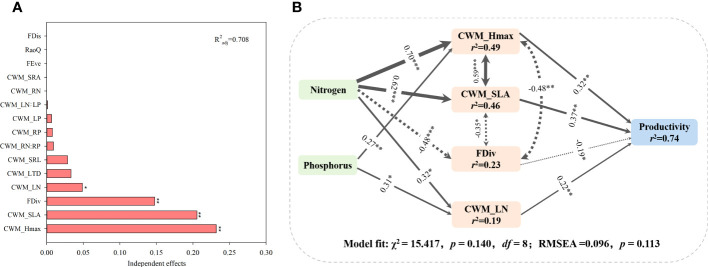
**(A)** Independent effects of functional diversity and CWM traits on productivity were evaluated on the basis of hierarchical partitioning. **(B)** Structural equation model (SEM) indicating the relationship between nutrient addition, CWM_H_max_, CWM_SLA, Fdiv, CWM_LN and community productivity (χ^2 = ^15.417, *df* =8, *p* = 0.140). Solid and dashed arrows represent significant positive and negative pathways at *p< 0.05; **p< 0.01; ***p< 0.001.

Based on the hierarchical partitioning canonical analysis, CWM traits and FD indices explained 70.8% of variations in grassland productivity (**Figure 9A**). The variables which explained the highest part of the variability were CWM_H_max_ (R^2 = ^0.232, *p*< 0.01), followed by CWM_SLA (R^2 = ^0.206, *p*< 0.01), FDiv (R^2 = ^0.148, *p*< 0.01) and CWM_LN (R^2 = ^0.049, *p*< 0.05). The model explained 74% of total variations in grassland productivity (**Figure 9B**). According to the model, both N and P additions significantly promoted CWM_H_max_ and CWM_LN, and both traits positively affected grassland productivity. High grassland productivity was also positively associated with increasing CWM_SLA induced by N addition. However, N addition indirectly influenced grassland productivity through its negative effect on FDiv.

## 4 Discussion

### 4.1 Trait plasticity regulates species-specific responses to N and P addition

Plant response to N and P addition is manifested by the patterns of trait plasticity (Lin et al., 2020). This research revealed that the patterns of plasticity varied greatly among different plant traits and species (**Figures 2**, **3**). For instance, the plasticity indices of LN, LP, RN, and RP were greater than zero under N and P addition, indicating that the addition of both elements promoted the nutrient absorption capacity of plants. This research also revealed that the plasticity indices of RN and RP were higher than those of LN and LP, which is possibly due to (1) competition between the leaf and root for nutrients, (2) the root system having the advantage of being close to the nutrient source, and (3) fine roots being the most sensitive plant part to nutrient addition (Kleyer and Minden, 2015; Kou et al., 2018). Moreover, the plasticity indices of LN: LP and RN: RP were higher than zero in the N-only treatment, primarily owing to greater plasticity recorded for LN and RN under N addition than LP and RP. RN, RP, and SRL mostly performed greater plasticity than zero, which indicated the enhancement of nutrient absorption capacity after fertilization, and the greater plasticity of RN and RP compared with SRL and SRA (**Figure 3**), indicated that root nutrient concentrations are more plastic than root morphological traits under nutrient addition (Kramer-Walter and Laughlin, 2017). These results indicate that functional traits’ response to nutrients is trait-specific, and overall plant adaptation strategies can be formed by the coordination between leaf and root traits (Faucon et al., 2017).

Functional traits determine plant resource use and growth strategies under different fertilization levels (Funk et al., 2017). In this regard, plant strategies for nutrient uptake can be categorized into acquisitive and conservative strategies (Seastedt et al., 2020), with acquisitive plant species generally benefiting from higher functional trait plasticity (e.g., SLA and SRL) (Wang et al., 2018). *B. ischaemum* exhibited lower trait plasticity (e.g., SLA, LTD, SRL and SRA), implying that this species was conservative species. Conservative perennial plant species have high resource utilization efficiency with low requirements; however, at higher levels of fertilization, they are at competitive disadvantages with acquisitive species (Gonzalez-Paleo and Ravetta, 2018). Thus the relative biomass of *B. ischaemum* increased and benefited from low fertilization levels, while decreased at higher levels of fertilization. While the relative biomass of *L. davurica* was higher in the P-only treatment and N25 combined with P, which was associated with higher RP, SRL, and SRA (**Figure 4**). This was likely owing to the positive impacts of P on the biological N_2_-fixation performance of this legume species (Chen et al., 2020). In comparison, in nutrient-rich environments, plants with acquisitive traits (e.g., greater SLA) usually grow faster and gain competitive advantages (Wang et al., 2018). As a result, *A. scoparia* and *A. sacrorum* performed greater relative biomass at high levels of N and P, indicating that these two species was acquisitive plant species characterized by higher functional trait plasticity and higher H_max_ and SLA (**Figure 4**). We also noted that both *A. scoparia* and *A. sacrorum* gained greater SLA at higher levels of N and P.

### 4.2 Variation of community functional structure following fertilization were mainly from ITV

Unravelling the specific roles of ITV and ST in community functional composition shifts is essential for understanding the community’s response to environmental changes (Delpiano et al., 2020). It has been suggested that higher adaptation abilities of plant species in response to fertilizer addition positively correlate with relative contributions of ITV to variations in community-level mean trait values (Lü et al., 2018). In contrast, the relative contributions of ST to improving species tolerance in changing environments are of minor importance (Kichenin et al., 2013). Our research revealed that ITV significantly contributed to variations in most plant traits under N and P addition. For instance, RP and SLA increased significantly at the community level with N and P addition, and their variability was primarily explained by ITV. This suggested that these traits are more sensitive to N and P addition. However, there were a few traits whose variations under nutrient addition were primarily driven by ST. For instance, LN and RN increased significantly at the community level with P addition, and their variation was mainly attributed to ST.

A positive covariance effect between ITV and ST for CWM traits exhibited synergistic responses of these traits following nutrient addition, whereas negative covariations indicate opposite directions of ITV and ST in trait selection (Lepš et al., 2011). For instance, we recorded positive covariations between ITV and ST for CWM_H_max_ under the interactive effects of N and P addition, suggesting that these effects reinforced each other, leading to an increase in H_max_ at the community level following the addition of both nutrient elements. On the other hand, we recorded negative covariations between ITV and ST for CWM_LN and CWM_LN: LP under N and P addition, suggesting there were contrasting trends through ST and ITV, leading to weakening responses a decreasing trend of these leaf traits at the community level following nutrient addition (Siefert and Ritchie, 2016).

This research showed that ITV primarily affected these variations of community functional composition under four years nutrient addition experiments that the results were consistent with that ITV played a pivotal role in community functional composition variability during short-term nutrient addition, while ST became more prominent during long-term fertilization Zhou et al. (2018). The dominant effect of ITV also revealed that neglecting the role of ITV in community variability leads to a great underestimation of community functional composition responses to changing environments Lepš et al. (2011). However, factors such as functional traits and spatial scale play a role in the explanatory contribution of ITV and ST (Zhou et al., 2018).

### 4.3 CWM traits and functional diversity together effect diversity and productivity

Since grassland productivity is a prominent indicator of vigorous grassland ecosystems and grassland restoration(Schaub et al., 2020; Yang et al., 2022a), evaluating the impact of nutrient-induced variations in community functional structure can inform us of grassland ecosystem functioning. CWM traits and functional diversity have primarily been used to unravel the relationship between functional traits and grassland ecosystem functioning (Wang et al., 2020). We found that CWM traits and functional diversity play a pivotal contrasting role in explaining variations in grassland productivity under nutrient addition (**Figure 9**). On one hand, increasing CWM_SLA and CWM_H_max_ after nutrient addition directly promoted community productivity. This is because CWM_SLA and CWM_H_max_ are essential factors in determining the competitive ability of species for light (Zheng and Ma, 2018). La Pierre and Smith (2015) also demonstrated that nutrient addition promoted rapid plant growth and high community productivity typically through greater SLA and height. High CWM_SLA is generally associated with an enhanced photosynthetic rate and rapid biomass production (Funk et al., 2017; Mao et al., 2017). Plant height is a crucial factor determining plant competitive abilities (Siefert and Ritchie, 2016; Lin et al., 2020; Liu et al., 2021). Tall species can capture and incest more light energy into biomass production (DeMalach et al., 2017).

On the other hand, N addition led to a reduction in FDiv. Low FDiv values indicate a low degree of niche differentiation and, thus, high competition for resources such as light and nutrients (Mouchet et al., 2010). One possible explanation is that nutrient addition favours species with highly competitive abilities (i.e., species with higher H_max_ and SLA), leading to excluding species with low competitive abilities (Turcotte and Levine, 2016; Read et al., 2017; Liu et al., 2021). The results of this research showed that CWM traits work in concert with functional diversity to influence grassland productivity; thus, it is essential to consider both trait-based metrics to understand the effect of environmental changes on grassland ecosystem functioning.

## 5 Conclusion

In this research, we conducted a four-year nutrient addition study to assess community functional structures and grassland productivity under N and P addition in a grassland ecosystem in the Loess Plateau. This study proved that aboveground (i.e., leaf) and belowground (i.e., root system) traits exhibit coordinated variations under N and P addition, with ITV predominantly driving the variations in community functional structures in response to nutrient addition. We also found that variations in CWM traits and functional diversity jointly affect the response of grassland productivity to nutrient addition. Future studies should involve further long-term studies and additional traits data under different environments in order to extend the finding of this research to broader regions.

## Data availability statement

The raw data supporting the conclusions of this article will be made available by the authors, without undue reservation.

## Author contributions

YY, ZC, BX and ZW planned and designed the research and guided the entire process of the study; CD, WL and RZ conducted the experiments and finished the material collection, YY, HG and ZW analyzed data, wrote and edited the manuscript. All authors contributed to the article and approved the submitted version.
