# Supplementary material for: Contrasting patterns of community-weighted mean traits and functional diversity in driving grassland productivity changes under N and P addition
Source: Front Plant Sci. 2023 Aug 15;14:1145709. doi: 10.3389/fpls.2023.1145709 (PMC10465162; doi:10.3389/fpls.2023.1145709)
Supplement: Supplementary file 1 [file DataSheet_1.docx]

**Supplementary Material**

**The relative contributions of ITV and species turnover**

The relative contributions of ITV and species turnover with variation in CWM traits and functional diversity were calculated following the method of Lepš et al. (2011), which is based on the sum of squares decomposition method. For this, we calculated three types of sums of squares (SS) for CWM traits and functional diversity values: total CWM traits and functional diversity values (SS*_specific_*), fixed CWM traits and functional diversity values (SS*_fixed_*), and intraspecific CWM traits and functional diversity values (SS*_intra_*). SS*_specific_* was calculated using the relative biomass and trait values of each species in a given subplot, including both species turnover and ITV effects. SS*_fixed_* was calculated using the relative biomass of each species in a given subplot and averaged trait values of each species across all subplots, including only the effects of species turnover. SS*_intra_* was calculated using the following formula: SS*_intra_* = SS*_specific_* - SS*_fixed_*. The covariation (SS*_cov_*) between ITV and species turnover was calculated as follows: SS*_cov_*= SS*_specific_* - SS*_fixed_* - SS*_intra_*. A positive correlation between the SS*_fixed_* and SS*_intra_* indicates that the SS*_specific_* will be higher than when species turnover and ITV effects are independent.

**References**

Lepš, J., F. de Bello, P. Šmilauer, and J. Doležal. 2011. Community trait response to environment: disentangling species turnover vs intraspecific trait variability effects. Ecography 34:856–863.

Figure S1 The distribution diagram of N and P addition treatments in the grassland community.


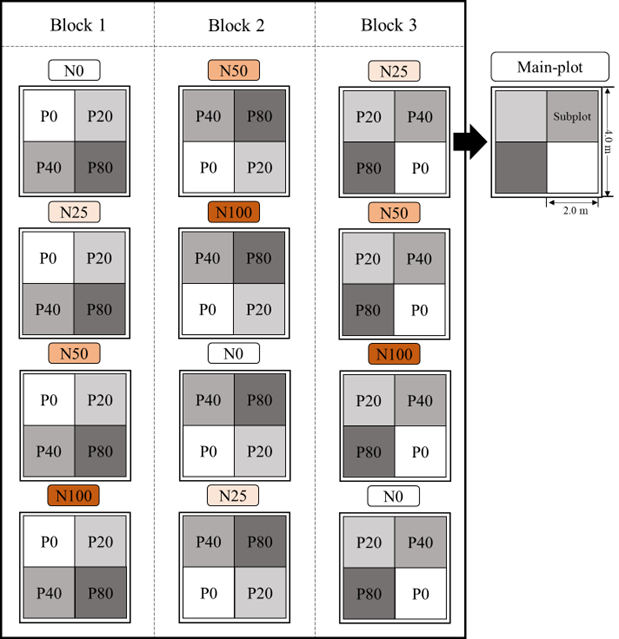


Table S1 Formula of functional diversity indices

| Index | Formula | Variable |
| --- | --- | --- |
| Functional  evenness | $\text{FEve}\text{ }\text{= }\frac{\sum_{\text{i}\text{=1}}^{\text{S}\text{-1}} \text{min}\left( \text{PEW}\text{i}\frac{\text{1}}{\text{S}\text{-1}} \right)\text{-}\frac{\text{1}}{\text{S}\text{-1}}}{\text{1-}\frac{\text{1}}{\text{S}\text{-1}}}$ | S is the number of species. *PEW_i_* is the partial weighted evenness of species *i*. |
| Functional divergence | $\text{FDiv}\text{ =}\frac{\text{2}}{\text{π}}\text{arctan}\left\{ \text{5×}\sum_{\text{i}\text{=1}}^{\text{N}} \left[ \left( \ln\text{C}\text{i}\text{-}\ln\text{x} \right)\text{2×}\text{A}\text{i} \right] \right\}$ | C_i_ is the character value for the *i*th functional character category. A_i_ is the proportional abundance of the *i*th functional character category. ln *x* is the abundance-weighted mean of the natural logarithm of character values for the categories. N is the number of traits. |
| Functional  dispersion | $\text{FDis}\text{ }\text{=}\text{ }\frac{\text{ }\sum\text{A}\text{j}\text{Z}\text{j}}{\sum\text{A}\text{j}}$ | A_j_ is the abundance of species *j*. Z_j_ is the distance of species *j* to centroidc. |
| Rao’s quadratic  entrop | $R\text{ao=}\sum_{\text{i}\text{=1}}^{\text{S}\text{-1}} \sum_{\text{j}\text{=}\text{i}\text{+1}}^{\text{S}} \text{D}\text{ij}\text{P}\text{i}\text{P}\text{j}$ | P_i_ and Z_j_ are the relative abundance of species *i* and species *j*. D_ij_ is the Euclidean distance between species *i* and species *j*. |

Table S2 Geographical and vegetation information of the three grassland communities before N and P additions in 2017.

| Grassland type | Restoration years (a) | Latitude and longitude | Altitude (m) | Aspects | Slope | Soil total N (TN) and total P (TP) content | Dominant species | Minor species |
| --- | --- | --- | --- | --- | --- | --- | --- | --- |
| Perennial grass community | 20 | 109°14′53″E  36°44′38″N | 1164 | E46°S | 9° | TN: 0.60 g kg^-1^  TP: 0.58 g kg^-1^ | *Bothriochloa ischcemum* (Linn.) Keng.  *Stipa bungeana* Trin.  *Lespedeza davurica* (Laxm.) Schindl. | *Artemisia sacrorum* Ledeb*.*  *Potentilla tanacetifolia* Willd. ex D.F.K.Schltdl., *Artemisia scoparia* (Waldst. & Kit.) Pamp. |

Table S3 The description of the six common species.

| Species | Family | Life history | Height |
| --- | --- | --- | --- |
| *Bothriochloa ischcemum* (L.) Keng. | Gramineae | Perennial | Tall |
| *Artemisia sacrorum* Ledeb*.* | Asteraceae | Perennial | Tall |
| *Lespedeza davurica* (Laxmann) Schindler | Leguminosae | Perennial | Short |
| *Stipa bungeana* Trin. | Gramineae | Perennial | Short |
| *Potentilla tanacetifolia* Willd. ex D.F.K.Schltdl. | Rosaceae | Perennial | Short |
| *Artemisia scoparia* (Waldst. & Kit.) Pamp. | Asteraceae | Annual | Tall |

Table S4 Analysis of variance results (F values) for the effects of N addition (N), P addition (P) and their interactions on leaf and root traits and maximum plant height (H_max_). n.s., *, ** and *** indicated non-significant, significant at *p* < 0.05, 0.01 and 0.001, respectively.

| Factors | LN  (g/kg) | LP  (g/kg) | LN: LP | SLA  (cm^2^/g) | LTD  (g/cm^3^) | RN  (g/kg) | RP  (g/kg) | RN: RP | SRL  (cm/g) | SRA  (cm^2^/g) | H_max_  (cm) |
| --- | --- | --- | --- | --- | --- | --- | --- | --- | --- | --- | --- |
| N | 2.53^*^ | 4.67^**^ | 10.15^***^ | 2.19^n.s.^ | 2.89^*^ | 0.98^n.s.^ | 1.37^n.s.^ | 3.58^*^ | 2.90^*^ | 0.29^n.s.^ | 7.67^***^ |
| P | 0.16^n.s.^ | 25.27^***^ | 48.38^***^ | 2.25^n.s.^ | 2.67^*^ | 0.35^n.s.^ | 31.68^***^ | 37.99^***^ | 1.66^n.s.^ | 0.39^n.s.^ | 0.19^n.s.^ |
| N * P | 0.21^n.s.^ | 1.10^n.s.^ | 0.54^n.s.^ | 0.12^n.s.^ | 0.28^n.s.^ | 0.34^n.s.^ | 0.51^n.s.^ | 0.27^n.s.^ | 0.26^n.s.^ | 0.48^n.s.^ | 0.82^n.s.^ |

Note: Leaf N concentration (LN); leaf P concentration (LP); leaf N:P ratio (LN: LP); specific leaf area (SLA); leaf tissue density (LTD); root N concentration (RN); root P concentration (RP); root N:P ratio (RN: RP); specific root length (SRL); specific root surface area (SRA); maximum plant height (H_max_).Table S5 Average trait plasticity index of the six species across all N and P addition levels.

| Functional traits | *B. ischaemum* | *S. bungeana* | *L. davurica* | *A. sacrorum* | *P. tanacetifolia* | *A. scoparia* |
| --- | --- | --- | --- | --- | --- | --- |
| LN | 0.16±0.02ab | 0.12±0.02bc | 0.07±0.01c | 0.12±0.02bc | 0.23±0.04a | 0.20±0.03a |
| LP | 0.28±0.05b | 0.40±0.06ab | 0.29±0.03b | 0.32±0.06b | 0.44±0.06ab | 0.47±0.04a |
| LN: LP | 0.27±0.04b | 0.42±0.05a | 0.24±0.03b | 0.31±0.05ab | 0.37±0.05ab | 0.35±0.05ab |
| SLA | 0.12±0.02c | 0.35±0.02a | 0.26±0.03c | 0.13±0.02b | 0.22±0.02b | 0.36±0.02a |
| LTD | 0.09±0.02e | 0.49±0.02a | 0.21±0.02d | 0.12±0.02e | 0.33±0.04c | 0.40±0.02b |
| RN | 0.27±0.03a | 0.29±0.03a | 0.10±0.01b | 0.30±0.04a | 0.26±0.04a | 0.37±0.06a |
| RP | 0.37±0.05bc | 0.47±0.05b | 0.51±0.05ab | 0.30±0.05c | 0.61±0.05a | 0.24±0.03c |
| RN: RP | 0.28±0.04b | 0.31±0.04b | 0.49±0.05a | 0.27±0.04b | 0.49±0.07a | 0.30±0.05b |
| SRL | 0.07±0.01d | 0.22±0.03c | 0.19±0.03c | 0.18±0.03c | 0.31±0.05b | 0.42±0.03a |
| SRA | 0.11±0.02cd | 0.18±0.03bc | 0.08±0.02d | 0.15±0.03bcd | 0.33±0.03a | 0.22±0.03b |

**Table S6** Relative biomass (%) of the six species under different N and P addition additions (mean ± s.e.; n = 3).

| N addition | P addition | *B. ischaemum* | *S. bungeana* | *L. davurica* | *A. sacrorum* | *P. tanacetifolia* | *A. scoparia* | Other species |
| --- | --- | --- | --- | --- | --- | --- | --- | --- |
| N0 | P0 | 47.30±10.54 | 0.78±0.78 | 11.70±2.34 | 6.70±5.99 | 11.36±8.94 | 5.39±2.23 | 16.76±3.58 |
|  | P20 | 24.78±11.75 | 3.95±3.95 | 40.41±9.92 | 0.33±0.33 | 3.60±2.30 | 5.64±2.13 | 21.30±3.48 |
|  | P40 | 9.96±4.39 | 2.25±2.25 | 46.83±5.47 | 0.00±0.00 | 12.59±4.19 | 8.27±2.21 | 20.10±2.30 |
|  | P80 | 19.42±2.46 | 6.53±4.17 | 47.78±1.70 | 1.46±1.46 | 4.04±2.45 | 4.44±1.43 | 16.33±1.39 |
| N25 | P0 | 36.88±6.98 | 16.39±2.30 | 10.70±2.61 | 6.67±3.46 | 8.48±6.26 | 2.21±0.91 | 18.66±4.85 |
|  | P20 | 50.09±4.51 | 8.03±1.98 | 16.43±0.92 | 7.48±6.55 | 5.61±2.30 | 2.96±1.39 | 9.40±5.11 |
|  | P40 | 27.71±9.49 | 6.09±0.40 | 21.59±0.73 | 11.45±6.67 | 4.57±2.80 | 10.22±4.67 | 18.36±5.89 |
|  | P80 | 25.73±14.35 | 10.28±1.02 | 24.16±12.04 | 10.74±6.58 | 4.68±4.68 | 4.63±2.25 | 19.78±0.90 |
| N50 | P0 | 26.16±8.39 | 27.54±11.68 | 9.36±5.15 | 14.03±6.62 | 3.03±0.83 | 6.59±2.94 | 13.29±0.59 |
|  | P20 | 28.79±10.35 | 11.12±6.62 | 19.06±2.71 | 13.63±6.88 | 0.21±0.21 | 11.68±6.09 | 15.51±2.61 |
|  | P40 | 29.75±10.73 | 8.11±2.47 | 23.07±2.85 | 5.68±5.68 | 3.58±1.30 | 8.68±5.30 | 21.13±1.37 |
|  | P80 | 31.69±7.47 | 14.61±3.75 | 15.50±3.91 | 6.86±1.80 | 3.65±1.88 | 10.78±1.67 | 16.92±3.45 |
| N100 | P0 | 25.80±8.19 | 26.05±19.35 | 12.93±6.31 | 6.72±4.36 | 0.96±0.62 | 6.07±4.91 | 21.49±2.73 |
|  | P20 | 14.54±4.40 | 4.65±2.16 | 2.80±1.28 | 38.38±7.09 | 3.68±0.33 | 12.03±6.18 | 23.91±3.90 |
|  | P40 | 20.03±3.30 | 6.07±2.19 | 7.96±1.62 | 19.90±3.96 | 3.37±1.73 | 29.33±8.23 | 13.33±3.22 |
|  | P80 | 18.51±2.46 | 3.70±0.79 | 4.19±0.78 | 35.70±4.44 | 1.49±1.12 | 19.88±2.23 | 16.53±2.31 |
| N | | *(9.32) | n.s. | ***(9.43) | *(11.55) | n.s. | *(7.75) | n.s. |
| P | | * (9.13) | * (8.67) | **(7.23) | n.s. | n.s. | *(5.61) | n.s. |
| N * P | | * (27.03) | n.s. | *(14.70) | *(15.34) | n.s. | n.s. | n.s. |

Note: Data in brackets are LSD values among different N and P addition treatments (*p* ≤ 0.05). ^*^, ^**^ and ^***^ indicate statistically significantly different at *p* ≤ 0.05, *p* ≤ 0.01, and *p*≤ 0.001, respectively. ns, means no significant difference. Same as following.
